# Prior Negative Experience Biases Activity of Medial Amygdala during Interstrain Social Engagement in Male Rats

**DOI:** 10.1523/ENEURO.0288-24.2024

**Published:** 2024-09-16

**Authors:** Alexandra C. Ritger, Nimah M. Rasheed, Mallika Padival, Nicole C. Ferrara, J. Amiel Rosenkranz

**Affiliations:** ^1^Department of Foundational Sciences and Humanities, Discipline of Neuroscience, Rosalind Franklin University, North Chicago, Illinois 60064; ^2^Department of Foundational Sciences and Humanities, Discipline of Cellular & Molecular Pharmacology, Rosalind Franklin University, North Chicago, Illinois 60064; ^3^Center for Neurobiology of Stress Resilience and Psychiatric Disorders, Rosalind Franklin University, North Chicago, Illinois 60064

**Keywords:** fiber photometry, medial amygdala, rat, social stress

## Abstract

Social recognition is an essential part of social function and often promotes specific social behaviors based on prior experience. Social and defensive behaviors in particular often emerge with prior experiences of familiarity or novelty/stress, respectively. This is also commonly seen in rodents toward same-strain and interstrain conspecifics. Medial amygdala (MeA) activity guides social choice based on age and sex recognition and is sensitive to social experiences. However, little is known about whether the MeA exhibits differential responses based on strain or how this is impacted by experience. Social stress impacts posterior MeA (MeAp) function and can shift measures of social engagement. However, it is unclear how stress impacts MeAp activity and contributes to altered social behavior. The primary goal of this study in adult male Sprague Dawley rats was to determine whether prior stress experience with a different-strain (Long–Evans) rat impacts MeAp responses to same-strain and different-strain conspecifics in parallel with a change in behavior using in vivo fiber photometry. We found that MeAp activity was uniformly activated during social contact with a novel same-strain rat during a three-chamber social preference test following control handling but became biased toward a novel different-strain rat following social stress. Socially stressed rats also showed initially heightened social interaction with novel same-strain rats but showed social avoidance and fragmented social behavior with novel different-strain rats relative to controls. These results indicate that heightened MeAp activity may guide social responses to novel, threatening, rather than non-threatening, social stimuli after stress.

## Significance Statement

We are challenged daily to interpret social information and decide on appropriate behaviors based on that information. Social decisions often favor conspecifics, especially those that are most similar, and this is further enhanced after stressful experiences. While this preference is conserved across mammalian species, its neural substrates are not known. We found that activity of the rodent posterior medial amygdala (MeAp), a brain region critical for early processing of social cues, distinguishes same- and different-strain conspecifics and responds differently to same-strain cues in the presence of a threatening different-strain conspecific. These results provide novel insight into how social threats redirect the encoding of social stimuli and can help explain how individuals balance different types of social information to direct behavior.

## Introduction

Social stressors contribute to the development and maintenance of several neuropsychiatric disorders (Paykel, 1978; Kessler et al., 1985; Turner, 1995; Heim et al., 1997; Monroe et al., 2009; Cunliffe, 2016). In turn, neuropsychiatric disorders produce a variety of effects on social behavior, including social avoidance and social anhedonia (McMakin et al., 2012; Barkus and Badcock, 2019). These social deficits are detrimental to quality of life and can impede treatment. A better understanding of the neural circuits involved in social behaviors and the impact of social stress is necessary to develop a framework for how the social environment impacts social functioning, as well as to develop more effective and targeted therapies.

In rodents, social engagement depends on a balance between social motivation and innate caution toward conspecifics which is influenced by familiarity, prior experience, and degree of similarity (Queller, 1985; Schneeberger et al., 2012; Bourke, 2014; Van Cleve and Akçay, 2014; Dolivo and Taborsky, 2015; Wood et al., 2016). Rodents are more likely to display prosocial behavior toward familiar individuals of the same strain and approach unfamiliar conspecifics more cautiously (Langford et al., 2006; Kiyokawa et al., 2014; Burkett et al., 2016; Liu and Yuan, 2016; Li et al., 2018). These factors are highly sensitive to stress, which can dampen social engagement with an unfamiliar conspecific (Blanchard et al., 1993, Potegal et al., 1993; Blanchard et al., 1995; Meerlo et al., 1996) and lead to higher caution toward strains that were the source of prior stress (Buwalda et al., 2005; Golden et al., 2011; Ayash et al., 2020). While prior studies demonstrate a key role for prefrontal cortical and amygdala systems in social decision-making, the neurobiology of stress-driven changes in social strain preference is unclear (Bergan et al., 2014; Li et al., 2017; Meyza et al., 2017; Yao et al., 2017; Carrillo et al., 2019; Bartal et al., 2021; Wu et al., 2021; Kietzman et al., 2024).

The medial amygdala (MeA) has a strong connection with social behaviors (Newman, 1999; Choi et al., 2005; Hong et al., 2014; Wang et al., 2014; Shemesh et al., 2016; Li et al., 2017; Aleyasin et al., 2018) and is a candidate region to guide stress-driven changes in social behavior, as social stress impacts MeA brain volume, functional connectivity, and gene transcription (Pezawas et al., 2005; Brown et al., 2020; Chen et al., 2024). Resident–intruder stress in particular is an ethologically relevant social stressor in male rats (Blanchard and Blanchard, 1977; Chaouloff, 2013) that produces lasting behavioral and physiologic changes within the MeA (Berton et al., 2006; Hollis and Kabbaj, 2014; Munshi et al., 2020; Warren et al., 2020). Social attacks in this paradigm increase immediate early gene expression in the MeA (Martinez, 1998; Covington et al., 2005; Fekete et al., 2009; Fanous et al., 2010; Wohleb et al., 2011; Lkhagvasuren et al., 2014). The MeA, particularly the posterior division (MeAp), is positioned as a potential site to modulate the effect of social stress on social behavior (Kollack-Walker and Newman, 1995; Martinez, 1998; Veening et al., 2005; Morrison et al., 2012). However, it is unknown how a prior history of social stress impacts the MeA's response to social stimuli. The primary goal of this study is to determine how the MeAp responds to different-strain unfamiliar conspecifics compared with same-strain unfamiliar conspecifics and whether prior stressful experience with the different strain shifts the MeAp neuronal response.

## Materials and Methods

The Rosalind Franklin University of Medicine & Science (RFUMS) animal program is accredited by the Association for Assessment and Accreditation of Laboratory Animal Care (AAALAC), and all procedures were approved by the RFUMS Institutional Animal Care and Use Committee (IACUC) and followed the NIH Guide for the Care and Use of Laboratory Animals. Efforts were taken to limit pain and reduce the number of animals used.

### Animals

Adult male Sprague Dawley outbred rats were purchased from Envigo. Rats were postnatal day (P) 53–58 and weighed 271–381 g at the start of the experiment and P81–103 and 300–402 g at termination. Rats were housed two or three per cage (17 × 8.5 × 8″) in the RFUMS Biological Resource Facility, maintained at constant temperature (22°C), 12 h reverse light/dark cycle (lights off 8 A.M. to 8 P.M.), and with food and water available *ad libitum*. Upon arrival, rats were given 1 week to acclimate to the facility's housing conditions. Rat home cages were randomly assigned to social stress or control groups (control, *n *= 49 rats; social stress, *n *= 51 rats) with a between-subjects design. Sample size was calculated using a power analysis based on a power level of 0.8, a *p* value of <0.05, and an effect size based on preliminary data or previous studies. The same-strain conspecific rats used for Trial 1 of the social preference test were Sprague Dawley males of the same age (within 10 d) and weight (within 76 g) as experimental rats. Retired breeder male Long–Evans rats were purchased from Envigo and singly housed without enrichment in the same facility. All experimental procedures were performed during the dark cycle.

### Experimental timeline

**Figure 1.**
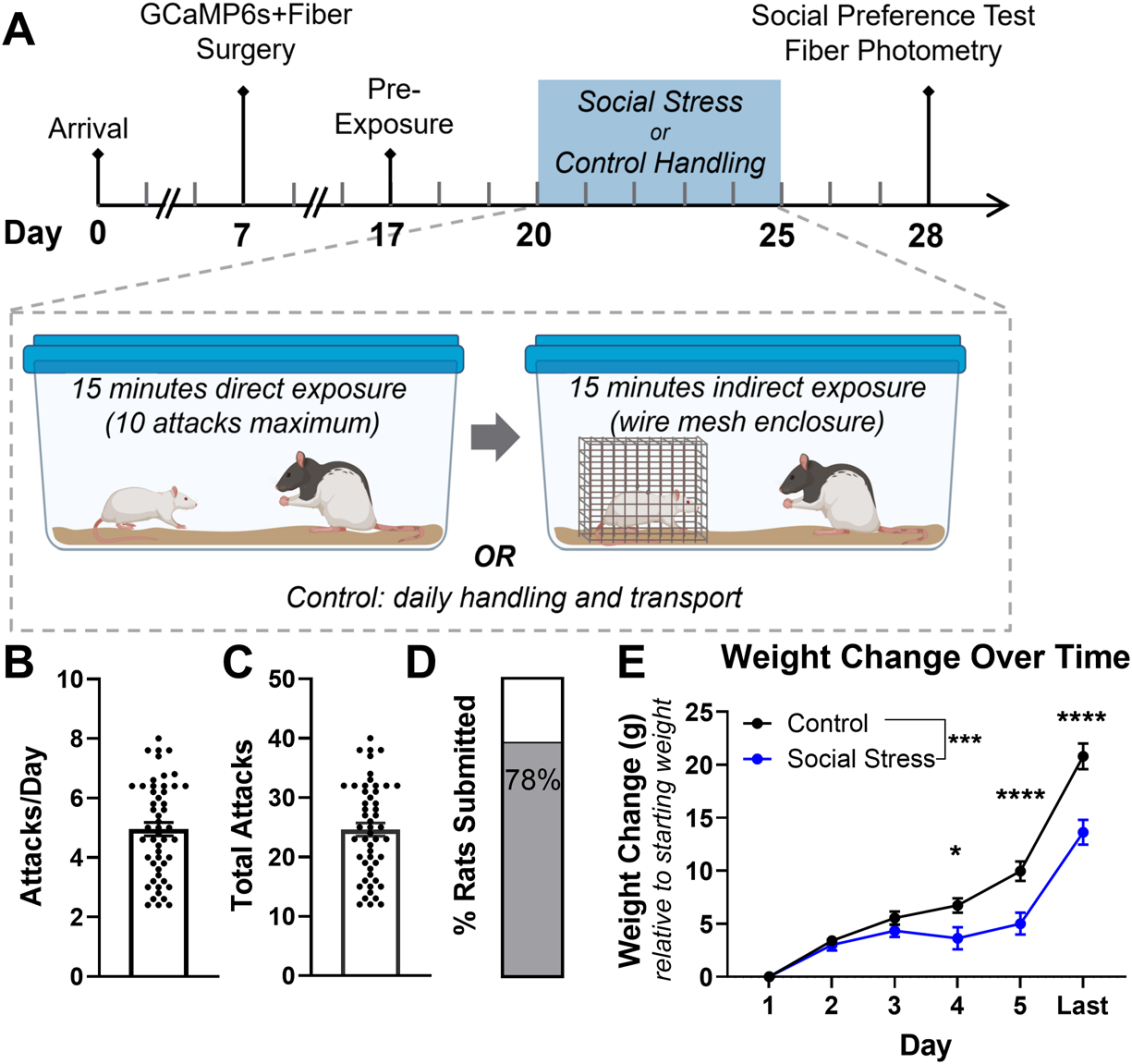
Experimental timeline and social stress. ***A***, Experimental timeline. Rats underwent surgery for GCaMP6s expression and fiber implantation followed by 2 weeks rest prior to social stress. Rats were pre-exposed to the social preference test (SoPT) apparatus and then exposed to 5 consecutive days of social stress or control procedures. Within 10 d of the last stress, rats underwent two trials of a SoPT with simultaneous fiber photometry recordings. ***B***, All rats in the social stress group experienced repeated attacks [average number of attacks per day and (***C***) total number of attacks]. ***D***, Most rats displayed at least one episode of submission posture. ***E***, Social stress reduced weight gain compared with control. Data shown as mean ± SEM. *p < 0.05, ***p < 0.001, ****p < 0.0001. Panel ***A*** created with BioRender.com.

After rats were acclimated to housing, they underwent survival surgery to produce expression of a genetically encoded calcium indicator (AAV5.Syn.GCaMP6s.WPRE.SV40; Chen et al., 2013) and implantation of a fiber-optic cannula aimed at MeAp. After 2 weeks of recovery, all rats were habituated to handling by experimenters and pre-exposed to the three-chambered social preference test (SoPT) apparatus at least 24 h prior to the first social stress (Fig. 1*A*). Rats then underwent 5 d of repeated social stress or control procedures. Within 10 d of social stress or control procedures, all rats underwent fiber photometry recordings during two trials of a SoPT. In Trial 1, rats were exposed to a novel same-strain rat and novel object. Trial 2 was conducted a few hours later and rats were exposed to a novel same-strain (Sprague Dawley) rat and a novel different-strain (Long–Evans) rat. At the end of the experiment, Sprague Dawley rats were deeply anesthetized and perfused, and brains were collected for histology.

### Survival surgery for AAV-GCaMP6s infusion and fiber implantation

Rats were injected with meloxicam (1 mg/kg in saline, s.c.) on the day of surgery and for 2 d after surgery to provide analgesia. All surgeries were performed using isoflurane gas anesthesia induced at 4% (in oxygen) and maintained at 1.8–2.5% throughout surgery, with adjustments in isoflurane percentage based on responsiveness to toe pinch. Aseptic techniques were used for surgery. Prior to incision, rats were placed in a stereotaxic frame, the scalp was cleaned with betadine and 70% ethanol, and 0.2 ml of lidocaine was injected subcutaneously. A burr hole was drilled in the skull superficial to the MeAp (A/P −3.0 mm, M/L ± 3.0 mm). Infusions were unilateral, and the infusion side was counterbalanced within and between groups. AAV viral vector [AAV5.Syn.GCaMP6s.WPRE.SV40 (Chen et al., 2013), viral prep # 100843-AAV5, RRID:Addgene_100843, titer ≥ 7 × 10^12^ vg/ml, Addgene] was injected into the MeAp (A/P −3.0 mm, M/L ± 3.0 mm, D/V −8.9 mm from skull) with a 10 µl glass Hamilton syringe with a 34-gauge needle (World Precision Instruments) via automated microinjector (UMP3T, World Precision Instruments) to a total volume of 500 nl (rate of 50 nl/min). The needle was left in place for an additional 10 min to allow for diffusion. Following the viral infusion, fiber-optic cannulae were implanted in the MeAp (A/P −3.2 mm, M/L ± 3.3 mm, D/V −8.4 mm from skull), 0.5 mm dorsal to the viral infusions. Cannulae were secured with four stainless steel screws, a thin layer of C&B Metabond adhesive luting cement (Parkell) and dental acrylic cement. Rats were allowed to recover for 2 weeks prior to social stress or control procedures and >3 weeks before photometry recordings to allow for adequate GCaMP6s expression.

### Resident–intruder social stress

Male rats were subjected to either 5 consecutive days of resident–intruder stress with a male retired breeder Long–Evans rat or control handling (Miczek, 1979; Hollis and Kabbaj, 2014; Munshi et al., 2022). Intruder rats (Sprague Dawley experimental subjects) were placed into a transport cage (12 × 6.5 × 5 in, clear polycarbonate) and transported to a separate procedure room for resident–intruder social stress. In that room, intruder rats were placed inside the home cage of a resident rat (Long–Evans, aggressor) for a stress session that included two contiguous phases. During the first phase (physical attack phase), resident–intruder pairs were allowed to freely interact for 15 min or until one of the following occurred: the intruder rat submitted [exhibited supine posture and freezing (Blanchard and Blanchard, 1977; Ruis et al., 1999; Hollis and Kabbaj, 2014)], the intruder rat was attacked 10 times, or 5 min passed with no attacks. After these 15 min (or after one of the above conditions occurred), the second phase began (nonphysical stress phase). During this 15 min second phase, the rats were physically separated by placing a smaller wire mesh cage over the intruder rat inside the resident's home cage, and the rats were not able to interact physically but continued to be exposed to visual, auditory, and olfactory cues from the other rat. Following the second phase, intruder rats were returned to their home cages. Intruder rats were exposed to a different resident rat for each session. Two or three rats individually underwent social stress in one room at a time under constant supervision by a trained experimenter. Rats that were injured were immediately removed from the resident's cage and examined. Rats with significant injuries (e.g., a large bite wound) were removed from the study. Control rats were placed in a transport cage for the same amount of time in the colony room. The number of attacks, latency to the first attack, time exposed to the stressor, whether submission occurred, latency to submission, and weight of the rat were measured daily.

While the effects of repeated social stress persist for several weeks (Hollis and Kabbaj, 2014; Munshi et al., 2020), we conservatively conducted all subsequent behavioral and photometry experiments within 10 d of the last stress session. Following social stress, all experimenters were blind to the treatment condition of the rats for the remainder of the experiment, including behavioral testing, fiber photometry, histology, and data analysis.

### Social preference test

**Figure 2.**
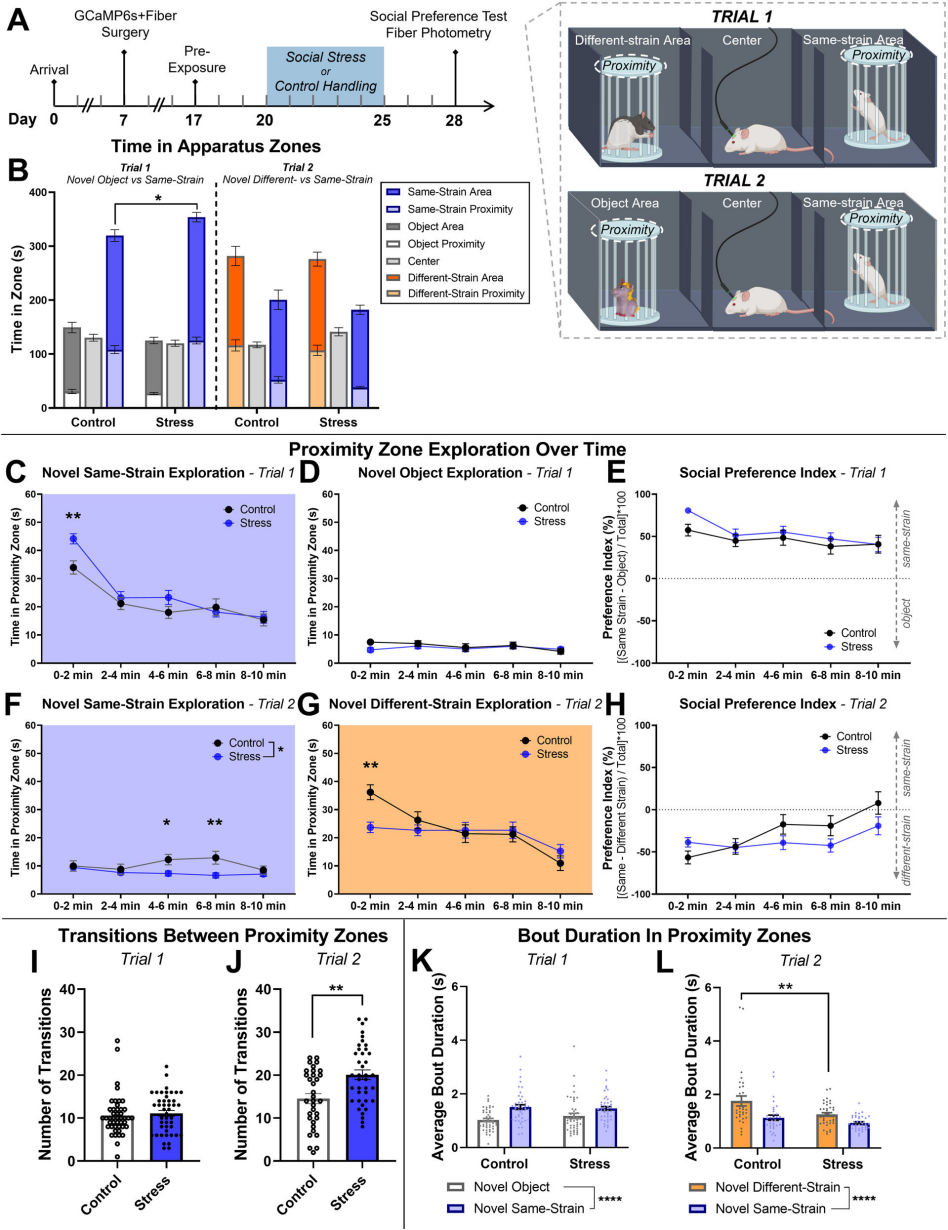
Social stress altered social behavior. ***A***, Experimental timeline and description of apparatus zones for Trial 1 (novel same-strain rat vs novel object) and Trial 2 (novel same-strain rat vs novel different-strain rat). The “proximity” zone consisted of the space immediately surrounding the cage (dotted line), and the “area” zone included the entire third of the apparatus (labelled). ***B***, Left, During Trial 1, both social stress and control groups spent more time in the novel same-strain rat area than the novel object area or center; however, socially stressed rats spent more time in the novel same-strain rat area compared with controls. ***B***, Right, During Trial 2, both groups spent more time in the different-strain rat area than the novel same-strain rat area or center, but there were no differences between stress and control groups. ***C***, In Trial 1, socially stressed rats spent more time investigating in the novel same-strain rat proximity zone at the beginning of the test relative to control and (***D***) did not show differences in time spent in the novel object proximity zone. ***E***, Socially stressed and control rats showed similar social preference over the course of Trial 1. ***F***, In Trial 2, socially stressed rats avoided spending time in the novel same-strain rat proximity zone compared with controls. ***G***, Socially stressed rats also avoided spending time in the novel different-strain rat proximity zone at the beginning of the test relative to controls. ***H***, These results were also reflected in the preference index in Trial 2, where both groups initially spent more time with the novel different-strain rat than the novel same-strain rat, but control rats slowly regained a preference for the novel same-strain rat over time while stressed rats did not (significant condition × time interaction). ***I***, Socially stressed and control rats showed a similar number of transitions between stimuli in Trial 1. ***J***, In Trial 2, socially stressed rats showed significantly more transitions between stimuli than control rats. We measured the amount of time spent with each stimulus rat to see if a high rate of transitions was associated with shorter bout duration. ***K***, In Trial 1, the average bout duration was similar between socially stressed and control rats while in both the novel same-strain rat proximity zone and the novel object proximity zone. ***L***, In Trial 2, the average bout duration was similar between social stress and control groups in the novel same-strain rat proximity zone. However, socially stressed rats had shorter average bout durations while in the novel different-strain rat proximity zone compared with controls, suggesting that socially stressed rats show increased social behavior fragmentation in the presence of a novel threat. Data shown as mean ± SEM. *p < 0.05, **p < 0.01. Panel ***A*** created with BioRender.com. Panel ***B*** bars are superimposed, as the “proximity” zone is included within the “area” zone.

The social preference test (Toth and Neumann, 2013), where an experimental rat chooses to interact with a novel object or novel conspecific that are contained within cylinders, allows analysis of social behaviors initiated fully by the experimental rat instead of conspecific. In addition, prior studies indicate that, even after social stress, rats display preference for social compared with nonsocial investigation (Lukas et al., 2011; Nyuyki et al., 2012). This is useful for our purposes because our goal was to measure MeAp activity during social investigation, which would not be feasible if rats became fully avoidant of social interaction after stress. This social preference test was performed in a three-chamber apparatus with an open top (100 × 45 × 40 cm, Maze Engineers). The chamber was divided into three compartments (center 25 × 45 × 40 cm) with a 13 cm opening in the center between each compartment, so the rat attached to the fiber-optic patch cord could freely pass between chambers. In two diagonally opposite corners, cylindrical cages (20 × 20 × 30 cm) with clear plexiglass bars were placed next to the walls. The zone immediately surrounding the cylindrical cage was defined as the “proximity” interaction zone, while each (larger) third of the apparatus was defined as the “area” (Fig. 2*A*). The apparatus was cleaned between each rat with 70% ethanol.

Experimental rats were handled to acclimate to gentle restraint for 2 consecutive days. On the following day (and at least 24 h prior to the first social stress/control handling session), rats were pre-exposed to the empty SoPT apparatus with the photometry patch cord attached. On the photometry recording day, immediately prior to the social preference test, rats underwent test-day habituation to the apparatus without novel objects/conspecifics for 10 min. During Trial 1 of the social preference test (novel same-strain rat vs novel object), a novel same-strain (Sprague Dawley) rat of the same age (within 10 d), sex, and weight (within 76 g) was placed in one cage, and a novel plastic toy (novel object) of similar size to the novel same-strain rat was placed in the opposite cage. The novel same-strain rat had been allowed to habituate to this environment for 5–10 min at least 24 h prior to the behavioral test. The experimental rat was allowed to move freely throughout the apparatus and interact with either stimulus for 10 min while attached to the fiber photometry patch cord. Trial 2 of the social preference test was conducted with another novel same-strain rat and a novel different-strain (Long–Evans) rat (instead of a novel object) in opposite cages. Trial 2 was conducted 1–4 h after Trial 1, with the exception of three control rats that were tested 21 h after Trial 1. The location of the novel same-strain rat and object (or novel different-strain rat) were counterbalanced both within and between subjects throughout the experiment. Behavior videos were automatically recorded, and the time spent within each interaction zone was automatically detected by tracking the rat's head and posture using ANY-maze software (version 6.34, Stoelting). The time spent in each zone, the number of entries to the zone, and the time oriented toward each stimulus (within 30°) were calculated. We also analyzed the amount of time spent in each zone in 2 min bins across the 10 min test, to capture changes over time. A preference index was calculated by subtracting time in the novel object proximity zone (in Trial 1) or novel different-strain rat proximity zone (in Trial 2) from the time near the novel same-strain rat proximity zone. This number was then divided by the total time investigating either proximity zone and is displayed as a percent. A positive preference index indicates that the rat spent more time engaging with the novel same-strain rat, and a negative preference index indicates that the rat spent more time investigating the novel object (Trial 1) or novel different-strain rat (Trial 2).

**Trial 1**:

$$\mathrm{Preference}\;\mathrm{index}=\frac{\mathrm{Novel}\;\mathrm{Same}\;\mathrm{Strain}\;\mathrm{Time}-\mathrm{Novel}\;\mathrm{Object}\;\mathrm{Time}}{\mathrm{Total}\;\mathrm{Investigation}\;\mathrm{Time}}\;\times 100.$$

**Trial 2**:

$$\mathrm{Preference}\;\mathrm{index}=\frac{\mathrm{Novel}\;\mathrm{Same}\;\mathrm{Strain}\;\mathrm{Time}-\mathrm{Novel}\;\mathrm{Different}\;\mathrm{Strain}\;\mathrm{Time}}{\mathrm{Total}\;\mathrm{Investigation}\;\mathrm{Time}}\;\times 100.$$

Rats have a natural preference for conspecifics compared with objects (Toth and Neumann, 2013), so a reduced preference index in Trial 1 represents social avoidance. We also calculated the number of times the experimental rat passed between the same-strain rat proximity zone and the novel object proximity zone (or novel different-strain rat proximity zone) and reported this as the total number of transitions between stimuli, as well as the number of transitions in 2 min bins.

We used Behavior Ensemble and Neural Trajectory Observatory (BENTO; Segalin et al., 2021), to identify direct nose–nose contact between the experimental rat and the stimulus rat and the MeAp Δ*F*/*F* activity aligned to those events. The frequency and duration of nose–nose contact between the rats was then hand-scored by a trained, blinded experimenter using free, open-source Behavioral Observation Research Interactive Software (BORIS; Friard and Gamba, 2016). To be conservative, we only scored direct nose–nose contact between the rats when it was clearly visible on video. The novel different-strain (Long–Evans) rats periodically attempted to bite or paw strike the experimental rat through the bars of the cage, and the experimental rats occasionally displayed startle behaviors during these episodes (sudden backing away from the cage and freezing). The onset time and frequency of these events were noted for later analysis.

### Fiber photometry

Fiber photometry recordings utilized the Fiber Photometry System from Doric Lenses and Tucker-Davis Technologies (RZ10X Expanded Lux10 Processor). A calcium-dependent signal (465 nm) and a calcium-independent (Lerner et al., 2015) isosbestic control signal (405 nm) were controlled through a light-emitting diode (LED) driver to allow for external modulation through a computer. The LED inputs both fed into a Mini Cube (Doric Lenses) via 400 µm patch cords with a 10% attenuation filter. From the Mini Cube, a patch cord allowed the rat to be connected to the fiber-optic cannula for local 465 and 405 light delivery. A pigtailed 1 × 1 fiber-optic rotary joint (Doric Lenses) allowed the cord to twist freely while the rat was connected. Patch cords were prebleached (2 mW) for ∼1 h prior to all recordings to ensure readings with low autofluorescence (Simone et al., 2018). Light intensity was maintained across behavioral sessions at 10 µW (Choi et al., 2019) and measured using an optical power meter (Power Meter PM100D, Thorlabs). This intensity was measured through the patch cord at the beginning and end of each recording day to ensure that light intensities through the fiber tip were within a 5–15 µW range during recording sessions. Synapse software (build 95-43524P, Tucker-Davis Technologies) was used to collect data and control the modulated 465 (210 Hz) and 405 (330 Hz) signals and demodulated, low-pass filtered (3 Hz) signals in real time through the RZ10X processor. Synapse software and the RZ10X processor were connected to video input for the recording of behavioral events. The Δ*F*/*F* signal was time locked to the behavior data by passing a TTL signal from ANY-maze (version 6.34, Stoelting) to Synapse for every frame captured in the video (30 frames/s).

Data analysis was done using MATLAB (MathWorks). We downsampled the raw 465 and 405 signals from 1,017.3 Hz to the ANY-maze video frame rate (30 Hz) by averaging 33 samples (∼33 ms) after the video frame timestamp based on the TTL input, so that each video frame aligned with one Δ*F*/*F* value. The 405 signal was then detrended using polynomial curve fitting to the 465 signal. Delta *F*/*F* (Δ*F*/*F*) values were calculated by subtracting the detrended 405 signal from the 465 signal and dividing by the detrended 405 signal to remove motion artifact (Lerner et al., 2015). Δ*F*/*F* values were normalized with a *z*-score [(Δ*F*/*F* − mean)/SD] to compare signals across recording sessions and between rats. The average Δ*F*/*F z*-score and area under the curve were calculated during the time spent exploring the novel same-strain rat, the novel object, the novel different-strain rat, and the center zone, as well as during the baseline recording in the empty apparatus (see Fig. 2*A* for zone definitions). Average Δ*F*/*F* in each zone was compared within subjects (e.g., during same-strain rat and object exploration) as well as between subjects (after social stress or control). We also calculated the relative MeAp Δ*F*/*F* activity in the social compartments by subtracting the activity while in the novel object proximity zone from the activity while in the novel same-strain rat proximity zone and compared this relative value across stress conditions. This calculation was also done for Trial 2 by subtracting the activity while in the novel different-strain rat proximity zone from the activity while in the novel same-strain rat proximity zone. The 10 min test was also divided into 2 min bins and the average Δ*F*/*F* was calculated in each zone, to analyze changes in activity across time. We also aligned the fiber photometry data with the onset and offset of social interaction epochs with a heat map to visualize MeAp activity proximal to social behavior (Gunaydin et al., 2014; Li et al., 2017).

Area under the curve was calculated for all nose–nose and startle events in each group by using trapezoidal numerical integration of MeAp Δ*F*/*F* activity at every data point according to the downsampled rate. The time period for AUC analysis was chosen qualitatively based on the area of the relative increase in activity compared with baseline (0–5 s postevent for nose–nose contact and −2.5 to 5 s postevent for startle behavior). Nose–nose events were only included in the analysis if they lasted for >1 s, and all startle events were included.

### Histology

**Figure 3.**
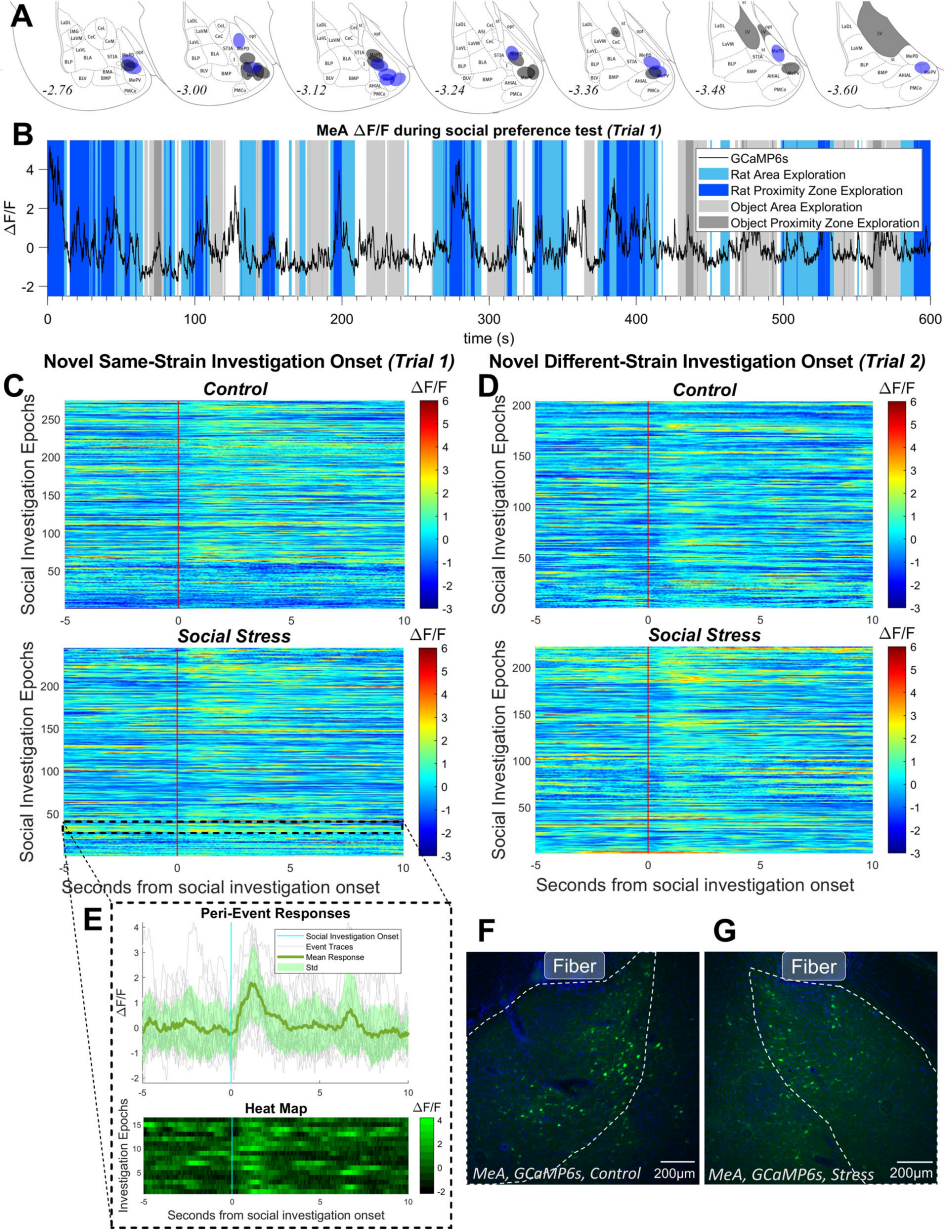
Histology and sample fiber photometry traces. ***A***, Placements of GCaMP6s virus below fiber with A/P coordinates displayed as mm from the bregma based on a rat brain atlas (Paxinos and Watson, 2009). Placements from control rats marked in black and placements from socially stressed rats marked in blue. ***B***, Sample fiber photometry recording in the posterior medial amygdala (MeAp) from a control rat in Trial 1 (novel same-strain rat vs novel object). Periods of social investigation are overlaid in blue and periods of object investigation are overlaid in gray. Darker shades represent closer exploration in the “proximity” zone rather than the “area” zone. Periods of time with no color overlay represent time spent in the center zone. ***C***, Peri-event heat maps demonstrated that the MeAp had increased activity following entry (red line) to the novel same-strain rat proximity zone in Trial 1 in both control and socially stressed rats. ***D***, The MeAp was also activated after entry to the novel different-strain rat proximity zone in both control and socially stressed rats, though the social stress group appeared to show higher MeAp activity than controls. ***E***, Sample peri-event traces from one socially stressed rat are shown in inset. ***F***, Representative images of GCaMP6s viral expression in control and (***G***) socially stressed rats.

Upon completion of experiments, experimental rats were deeply and rapidly anesthetized with isoflurane gas. Rats were then transcardially perfused with 50 ml of 0.1 M phosphate-buffered saline (PBS) followed by 50 ml of 4% paraformaldehyde (PFA). Rats were decapitated and brains were extracted and fixed in 4% PFA overnight and then transferred into 0.1 M PBS until sectioning. Free-floating tissue sections (40 μm) were collected using a vibratome (Leica Microsystems) and mounted on charged slides, and coverslips were applied using mounting media containing DAPI (Fluoroshield with DAPI, Sigma-Aldrich). The locations of the fiber and viral green fluorescent protein (GFP) expression were verified using a rat brain atlas (Paxinos and Watson, 2009). All recording sites are shown in Fig. 3*A* and representative images of viral expression are shown in Figure 3*F* (control) and Figure 3*G* (stress).

### Data analysis and statistics

Data was analyzed using GraphPad Prism statistical software (version 8.0). The figures were created using Prism, Gravit Designer (CorelDRAW), and BioRender software. Data are represented as group averages with standard error of the mean (SEM). Statistically significant differences between groups were determined as *p* < 0.05. Data were tested for outliers (Grubbs’, alpha = 0.01), and if identified, outliers were removed and indicated in the Results. Between-subjects comparisons between social stress and control groups were analyzed with two-tailed unpaired *t* tests or Mann–Whitney *U* tests when the data was not normally distributed, based on a Kolmogorov–Smirnov test. Measures of social preference were compared within subjects (e.g., social vs object exploration) as well as between subjects (social stress vs control), with two-way mixed-effects model analyses of variance (ANOVAs) with Holm–Sidak's multiple-comparisons post hoc tests following significant main effects and interactions. Correlations were analyzed using simple linear regressions. Analyses across time were analyzed using two-way mixed-model analyses of variance (ANOVAs) with Holm–Sidak's multiple-comparisons post hoc tests following significant main effects and interactions. Details of statistical analyses for each comparison are provided in Table 1 and are referenced as superscript letters throughout the Results section.

**Table 1.** Statistical table

	Data structure	Type of test	Power (confidence interval)
a—average number of attacks/day 5.0 ± 0.22	Normal	No test	95% CI [4.2, 5.8]
b—average number of total attacks 24.6 ± 1.1	Normal	No test	95% CI [21.0, 28.0]
c—weight gained, main effect of condition (stress), *F*_(1,98) _= 11.6, *p *= 0.0010	Normal	Two-way mixed-effects model ANOVA	95% CI of difference [1.2, 4.4]
d—weight gained, Day 4, *p *= 0.022	Normal	Holm–Sidak's^a^ multiple-comparisons test	95% CI [0.16, 6.0]
e—weight gained, recording day, *p *< 0.0001	Normal	Holm–Sidak's multiple-comparisons test	95% CI [4.2, 10.1]
f—time spent in the novel same-strain rat or novel object area, condition × zone interaction, *F*_(2,264) _= 6.9, *p *= 0.0012	Normal	Two-way mixed-effects model ANOVA	95% CI of difference [10.5, 106.2]
g—time in the novel same-strain rat area, *p *= 0.011	Normal	Holm-Sidak's multiple-comparisons test	95% CI [−61.9,−6.2]
h—time spent in the novel same-strain rat or novel different-strain rat area, main effect of zone, *F*_(2,210) _= 74.1, *p *< 0.0001	Normal	Two-way mixed-effects model ANOVA	95% CI of difference [48.1, 127.0]
		(to obtain CI of difference between same-strain and different strain, reran without center zone)	
i—time in the novel same-strain rat proximity zone over time, condition × time interaction (T1), *F*_(4,352) _= 2.7, *p *= 0.031	Normal	Two-way mixed-effects model ANOVA	95% CI of difference [−16.7, −1.8]
			True CI interaction computation not possible because >2 (5) time points. As proxy, CI of interaction computed from data collapsed to T0-2 and T8-10
j—time in the novel same-strain rat proximity zone (T1), 0–2 min time point, *p *= 0.0067	Normal	Holm–Sidak's multiple-comparisons test	95% CI [−18.0, −2.0]
k—preference index over time (T1), main effect of time, *F*_(4,350) _= 4.8, *p *= 0.0009	Normal	Two-way mixed-effects model ANOVA	95% CI of difference [14.9, 41.8]
			True CI main effect computation not possible because >2 (5) time points. As proxy, CI of interaction computed from data collapsed to T0-2 and T8-10
l—time in the novel same-strain rat proximity zone over time (T2), main effect of condition, *F*_(1,70) _= 5.5, *p *= 0.022	Normal	Two-way mixed-effects model ANOVA	95% CI of difference [0.42, 5.3]
m—time in the novel same-strain rat proximity zone (T2), 4–6 min time point, *p *= 0.048	Normal	Holm–Sidak's multiple-comparisons test	95% CI [−0.13, 10.0]
n—time in the novel same-strain rat proximity zone (T2), 6–8 min time point, *p *= 0.0073	Normal	Holm–Sidak's multiple-comparisons test	95% CI [1.2, 11.4]
o—time in the novel different-strain proximity zone over time, main effect of time, *F*_(4,280) _= 21.2, *p *< 0.0001	Normal	Two-way mixed-effects model ANOVA	95% CI of difference [13.3, 20.4]
			True CI main effect computation not possible because >2 (5) time points. As proxy, CI of interaction computed from data collapsed to T0-2 and T8-10
p—time in the novel different-strain proximity zone over time, condition × time interaction, *F*_(4,280) _= 6.3, *p *< 0.0001	Normal	Two-way mixed-effects model ANOVA	95% CI of difference [9.7, 23.9]
			True CI interaction computation not possible because >2 (5) time points. As proxy, CI of interaction computed from data collapsed to T0-2 and T8-10
q—time in the novel different-strain rat proximity zone, 0–2 min time point, *p *= 0.0038	Normal	Holm–Sidak's multiple-comparisons test	95% CI [3.0, 22.0]
r—preference index over time (T2), main effect of time, *F*_(4,278) _= 9.1, *p *< 0.0001	Normal	Two-way mixed-effects model ANOVA	95% CI of difference [−59.2, −24.7]
			True CI main effect computation not possible because >2 (5) time points. As proxy, CI of interaction computed from data collapsed to T0-2 and T8-10
s—preference index over time (T2), condition × time interaction, *F*_(4,278) _= 3.0, *p *= 0.020	Normal	Two-way mixed-effects model ANOVA	95% CI of difference [−79.4, 10.5]
			True CI interaction computation not possible because >2 (5) time points. As proxy, CI of interaction computed from data collapsed to T0-2 and T8-10
t—number of transitions between novel same-strain and novel object proximity zones, *U *= 905, *p *= 0.39 (n.s.)	Non-normal	Mann–Whitney *U* test	Control 10.6 ± 0.7 transitions, 95% CI [9.2, 12.0]; stress 11.1 ± 0.7 transitions, 95% CI [9.7, 12.5]; sum of ranks [1,940, 2,155], n.s.
u—bout duration in the novel same-strain rat proximity zone and novel object proximity zone, main effect of apparatus zone, *F*_(1,88) _= 27.5, *p *< 0.0001	Normal	Two-way mixed-effects model ANOVA	95% CI of difference [−0.52 to −0.24]
v—number of transitions between same-strain and different-strain rat proximity zones, *U *= 386, *p *= 0.0032	Non-normal	Mann–Whitney *U* test	Control 14.5 ± 1.2 transitions, 95% CI [12.2, 16.9]; stress: 20.1 ± 1.1 transitions, 95% CI [17.8, 22.3]; sum of ranks [947, 1,681]
w—bout duration in the novel same-strain rat proximity zone and novel different-strain rat proximity zone, main effect of apparatus zone, *F*_(1,70) _= 20.2, *p *< 0.0001	Normal	Two-way mixed-effects model ANOVA	95% CI of difference [0.26, 0.68]
x—bout duration in the novel same-strain rat proximity zone and novel different-strain rat proximity zone, main effect of condition, *F*_(1,70) _= 11.0, *p *= 0.0015	Normal	Two-way mixed-effects model ANOVA	95% CI of difference [0.14, 0.56]
y—bout duration in the different-strain rat proximity zone, *p *= 0.0019	Normal	Holm–Sidak's multiple-comparisons test	95% CI [0.17, 0.84]
*z*—average MeAp Δ*F*/*F* while in the novel same-strain rat proximity zone compared with the novel object proximity zone, main effect of zone, *F*_(1,19) _= 55.5, *p *< 0.0001	Normal	Two-way mixed-effects model ANOVA	95% CI of difference [−0.95, −0.53]
A—average MeAp Δ*F*/*F* while in the novel same-strain proximity zone compared with novel different-strain rat proximity zone, condition × zone interaction, *F*_(1,18) _= 17.2, *p *= 0.0006	Normal	Two-way mixed-effects model ANOVA	95% CI of difference [0.39, 1.2]
B—average MeAp Δ*F*/*F* while in the novel different-strain rat proximity zone, *p *= 0.0031	Normal	Holm–Sidak's multiple-comparisons test	95% CI of difference [−0.82, −0.15]
C—average MeAp Δ*F*/*F* while in the novel same-strain rat proximity zone, *p *= 0.033	Normal	Holm–Sidak's multiple-comparisons test	95% CI of difference [−0.016, 0.65]
D—relative (same-strain–object) MeAp Δ*F*/*F*, *t*_(20) _= 0.53, *p *= 0.60 (n.s.)	Normal	Two-tailed unpaired *t* test	Control, 0.77 ± 0.12, 95% CI [0.51, 1.0]; stress, 0.67 ± 0.16, 95% CI [0.32, 1.0]; 95% CI of difference [−0.51, 0.30], n.s.
E—relative (same-strain–different-strain) MeAp Δ*F*/*F*, *t*_(18) _= 4.1, *p *= 0.0006	Normal	Two-tailed unpaired *t* test	Control: *n *= 10 rats, 0.47 ± 0.14, 95% CI [0.16, 0.79]; stress: *n *= 10 rats, (−0.33) ± 0.13, 95% CI [−0.63, −0.024]; CI of difference [−1.2, −0.39]
F—amount of time in nose–nose interaction with the novel same-strain rat (T1), *t*_(19) _= 0.47, *p *= 0.64 (n.s.)	Normal	Two-tailed unpaired *t* test	Control, 27.6 ± 2.9 s, 95% CI [21.2, 33.9]; stress, 25.2 ± 4.3 s, 95% CI [15.4, 34.9]; CI of difference [−13.1, 8.2], n.s.
G—amount of time in nose–nose interaction with the novel same-strain rat (T2), *t*_(19) _= 2.9, *p *= 0.010	Normal	Two-tailed unpaired *t* test	Control, 15.0 ± 3.3 s, 95% CI [7.5, 22.4]; stress, 5.4 ± 1.1 s, 95% CI [2.9, 7.9]; CI of difference [−16.6, −2.6]
H—amount of time in nose–nose interaction with the novel different-strain rat (T2), *p *= 0.24 (n.s.)	Normal	Two-tailed unpaired *t* test	Control, 10.4 ± 1.7 s, 95% CI [6.6, 14.2]; stress, 13.6 ± 2.0 s, 95% CI [9.1, 18.2]; CI of difference [−2.4, 8.8], n.s.
I—MeAp Δ*F*/*F*, novel same-strain rat, nose–nose contact and proximity zone (T1), main effect of location, *F*_(2,38) _= 76.7, *p *< 0.0001	Normal	Two-way mixed-effects model ANOVA	95% CI of difference [−0.77, −0.46]
J—MeAp Δ*F*/*F*, nose–nose contact novel same-strain rat and novel different-strain rat (T2), main effect of zone, *F*_(1,38) _= 3.7, *p *= 0.060 (n.s.)	Normal	Two-way mixed-effects model ANOVA	95% CI of difference [−0.016, 0.70], n.s.
K—MeAp Δ*F*/*F* after initiating nose–nose contact with novel same-strain rat (T1), *U *= 1691, *p *= 0.60 (n.s.)	Non-normal	Mann–Whitney *U* test	Control, 4.7 ± 0.6, 95% CI [3.4, 5.9]; stress, 4.3 ± 0.6, 95% CI [3.0, 5.5]; sum of ranks [3,973, 3,287], n.s.
L—MeAp Δ*F*/*F* after initiating nose–nose contact with novel same-strain rat (T2), *t*_(35) _= 0.67, *p *= 0.51 (n.s.)	Normal	Two-tailed unpaired *t* test	Control, 5.6 ± 0.8, 95% CI [4.1, 7.2]; stress, 6.7 ± 1.4, 95% CI [3.4, 9.9]; CI of difference [−2.1, 4.1], n.s.
M—MeAp Δ*F*/*F* after initiating nose–nose contact with novel different-strain rat (T2), *t*_(91) _= 2.8, *p *= 0.0065	Normal	Two-tailed unpaired *t* test	Control, 1.9 ± 0.4, 95% CI [1.1, 2.8]; stress, 4.0 ± 0.6, 95% CI [2.8, 5.2]; CI of difference [0.59, 3.5]
N—MeAp Δ*F*/*F* after initiation of defensive response (startle, T2), *t*_(44) _= 3.4, *p *= 0.0015	Normal	Two-tailed unpaired *t* test	Control, 1.1 ± 0.9, 95% CI [−0.8, 3.0]; stress, 5.6 ± 1.0, 95% CI [3.6, 7.6]; CI of difference [1.8, 7.1]

a T1 = Trial 1, T2 = Trial 2. Note: GraphPad Prism does not calculate CI for Holm–Sidak's multiple-comparisons test. Therefore, CI was calculated independently of Holm–Sidak's multiple-comparisons test.

## Results

### Social stress impaired weight gain in Experiment 1

All rats in the social stress group experienced repeated attacks (Fig. 1*B*; average number of attacks/day 5.0 ± 0.22, 95% CI [4.2, 5.8]^a^; Fig. 1*C*; average number of total attacks 24.6 ± 1.1, 95% CI [21.0, 28.0]^b^), and most rats displayed at least one episode of submission posture (Fig. 1*D*; 40/51 rats). To verify the efficacy of repeated social stress, we measured body weight change. Socially stressed rats gained less weight than controls throughout the experiment (Fig. 1*E*; main effect of condition, *F*_(1,98) _= 11.6, *p *= 0.0010; main effect of time, *F*_(5,490) _= 204.1, *p *< 0.0001; condition × time interaction, *F*_(5,490) _= 11.8, *p *< 0.0001, two-way mixed-effects model ANOVA)^c^, first observed on Day 4 (*p *= 0.022)^d^ and persisting to the photometry recording day (*p *< 0.0001, post hoc Holm–Sidak's multiple-comparisons test)^e^. The impaired weight gain indicates that the resident–intruder procedure under these conditions produced stress in these animals, consistent with prior studies (Warren et al., 2013; Hollis and Kabbaj, 2014).

### Social stress shifted social preference

To measure effects of social stress on preference for social interaction relative to nonsocial exploration, time spent in the novel same-strain rat or novel object area of the apparatus was analyzed in Trial 1 (see Fig. 2*A* for zone definitions). Social stress impacted time spent in the novel same-strain rat or novel object area of the apparatus [Fig. 2*B*, left; main effect of zone (object area vs center vs same-strain area), *F*_(2,264) _= 420.9, *p *< 0.0001; condition × zone interaction, *F*_(2,264) _= 6.9, *p *= 0.0012, two-way mixed-effects model ANOVA]^f^. Both groups spent more time in the novel same-strain rat area than the novel object area or center; however, socially stressed rats spent more time in the novel same-strain rat area than controls (*p *= 0.011, post hoc Holm–Sidak's multiple-comparisons test)^g^.

To measure the preference between a different-strain rat and same-strain rat, and how this is shifted if the different strain was a prior source of social stress, time spent in the novel same-strain rat or novel different-strain rat area of the apparatus was analyzed in Trial 2. In Trial 2, both groups spent more time in the area containing the novel different-strain rat relative to the novel same-strain rat area [Fig. 2*B*, right; main effect of zone (object area vs center vs same-strain area), *F*_(2,210) _= 74.1, *p *< 0.0001, two-way mixed-effects model ANOVA]^h^. Though the novel different-strain rat was confined inside a cage, we noted that the different-strain rat still occasionally attempted paw strikes and small bites through the gaps in the cage bars. The experimental rats generally responded by vocalizing and displaying freezing, stretch and attend postures, and slower movements. So, it is possible that despite the greater time spent in the novel different-strain rat zone, the behavioral preference might not discern whether choices are driven by the valence of the stimulus.

### Social stress changed social behavior dynamics over time

Analyses over time can detect changes in behavior that would otherwise be washed out over the course of a 10 min test. Rats exhibit significant changes in social motivation and social anxiety over time (Netser et al., 2020; Ferrara et al., 2022; Loh et al., 2023) that are not adequately detected when averaged together. Therefore, we analyzed the 10 min social preference test in 2 min bins to detect changes in stimulus investigation over time. For these measures, we looked at time in the “proximity” zone, to capture more specific changes in behavior than those that might be detected in the larger “area.”

In Trial 1 (novel same-strain rat vs novel object), we found that stressed rats spent more time in the novel same-strain rat proximity zone than controls at the beginning of the test (Fig. 2*C*; main effect of time, *F*_(4,352) _= 42.9, *p *< 0.0001; condition × time interaction, *F*_(4,352) _= 2.7, *p *= 0.031, two-way mixed-effects model ANOVA^i^; 0–2 min time point, *p *= 0.0067, post hoc Holm–Sidak's multiple-comparisons test^j^). There were no significant effects of condition (social stress or control) or on time in the novel object proximity zone (Fig. 2*D*). To determine whether the observed differences in social behavior over time were due to a relative preference for one stimulus over the other, we also analyzed the preference index across time. We found that in Trial 1 (novel same-strain rat vs novel object), the preference index was high (favored the novel same-strain rat) in both stress and control groups and decreased slightly across time (Fig. 2*E*; main effect of time, *F*_(4,350) _= 4.8, *p *= 0.0009, two-way mixed-effects model ANOVA)^k^; however, there were no differences between stress and control groups.

In Trial 2 (novel same-strain rat vs novel different-strain rat), we found that stressed rats spent less time in the novel same-strain rat proximity zone than controls, which is in marked contrast to Trial 1 [Fig. 2*F*; main effect of condition, *F*_(1,70) _= 5.5, *p *= 0.022, two-way mixed-effects model ANOVA^l^; 4–6 min time point (*p *= 0.048)^m^ and 6–8 min time point (*p *= 0.0073)^n^, post hoc Holm–Sidak's multiple-comparisons test]. This indicates that stressed rats are avoidant of novel same-strain rats, but only while in the presence of a different strain with which they had prior aversive experience. Both stress and control rats spent less time in the novel different-strain proximity zone over time, but this effect was different between the two conditions (Fig. 2*G*; main effect of time, *F*_(4,280) _= 21.2, *p *< 0.0001^o^; condition × time interaction, *F*_(4,280) _= 6.3, *p *< 0.0001^p^, two-way mixed-effects model ANOVA). Stressed rats spent less time in the novel different-strain rat proximity zone at the 0–2 min time point than controls (*p *= 0.0038, post hoc Holm–Sidak's multiple-comparisons test)^q^. This indicates that socially stressed rats are initially more avoidant of the different-strain rat than controls, as would be expected. This is consistent with stressed rats avoiding both the different-strain rat (with which they had prior aversive experience) and the same-strain rat compared with controls, but only if the different-strain rat is present.

The Trial 2 preference index favored the novel different-strain rat in both stress and control groups. However, the preference index increased over time toward the same-strain rat in the control group relative to the stressed group (Fig. 2*H*; main effect of time, *F*_(4,278) _= 9.1, *p *< 0.0001^r^; condition × time interaction, *F*_(4,278) _= 3.0, *p *= 0.020^s^, two-way mixed-effects model ANOVA). Based on the results described above, this difference in preference between social stress and control groups in Trial 2 is due to control rats shifting their preference from the different-strain rat to a more equal distribution between social stimuli, while stressed rats avoid both social stimuli but spent more time near the different-strain rat compared to the same-strain rat.

This pattern of more time spent in the proximity zone with a different-strain rat during the early parts of the assay may reflect uncertainty behavior, where rats spend more time sampling the social environment. Several studies have even shown paradoxical increases in social approach toward aggressive conspecifics following social stress (Nelson et al., 2010; Cumming et al., 2014; Boyson et al., 2016). To test whether the stressed rats showed more uncertainty behaviors, we measured the number of transitions between zones.

### Social stress increased the number of transitions between stimuli and shortened bout duration with a different-strain rat

Transitions between zones and social bout durations have been used to gauge rodent social sampling and uncertainty (Netser et al., 2017, 2020). In Trial 1, socially stressed and control rats had a similar number of transitions between stimuli (novel same-strain and novel object proximity zones; Fig. 2*I*; control: *n *= 45 rats, 10.6 ± 0.7 transitions, 95% CI [9.2, 12.0]; stress: *n *= 45 rats, 11.1 ± 0.7 transitions, 95% CI [9.7, 12.5]; *U *= 905, *p *= 0.39, Mann–Whitney *U* test)^t^. Both groups had a longer average bout duration in the novel same-strain rat proximity zone than the novel object proximity zone, but there was no difference between stress and control groups (Fig. 2*K*; main effect of apparatus zone, *F*_(1,88) _= 27.5, *p *< 0.0001, two-way mixed-effects model ANOVA)^u^.

In Trial 2, socially stressed rats transitioned between stimuli (same-strain and different-strain rat proximity zones) more times than controls (Fig. 2*J*; control: *n *= 33 rats, 14.5 ± 1.2 transitions, 95% CI [12.2, 16.9]; stress: *n *= 39 rats, 20.1 ± 1.1 transitions, 95% CI [17.8, 22.3]; *U *= 386, *p *= 0.0032, Mann–Whitney *U* test)^v^. There was also a significant difference in average bout duration between socially stressed and control rats in the novel same-strain rat proximity zone and novel different-strain rat proximity zone (Fig. 2*L*; main effect of apparatus zone, *F*_(1,70) _= 20.2, *p *< 0.0001^w^, main effect of condition, *F*_(1,70) _= 11.0, *p *= 0.0015^x^, two-way mixed-effects model ANOVA). Socially stressed rats had a shorter average bout duration in the different-strain rat proximity zone compared with controls (*p *= 0.0019, post hoc Holm–Sidak's multiple-comparisons test)^y^. Taken with the higher number of transitions, this indicates more fragmented social behavior by the stressed rats in the presence of a different strain with which they had prior aversive experience.

Altogether, the results demonstrate a persistent social preference for a novel same-strain rat compared with an object and a preference for investigating a novel different-strain rat that diminishes over time, but the amount of time engaged in social behavior and preference is sensitive to prior aversive experience with a different strain. This provides a unique set of conditions to test how the response of the MeAp to conspecifics is modified by prior stress and by the presence of a conspecific strain that was the cause of the prior stress.

### Social stress impacts MeAp activity during social exploration in a stimulus-dependent manner

**Figure 4.**
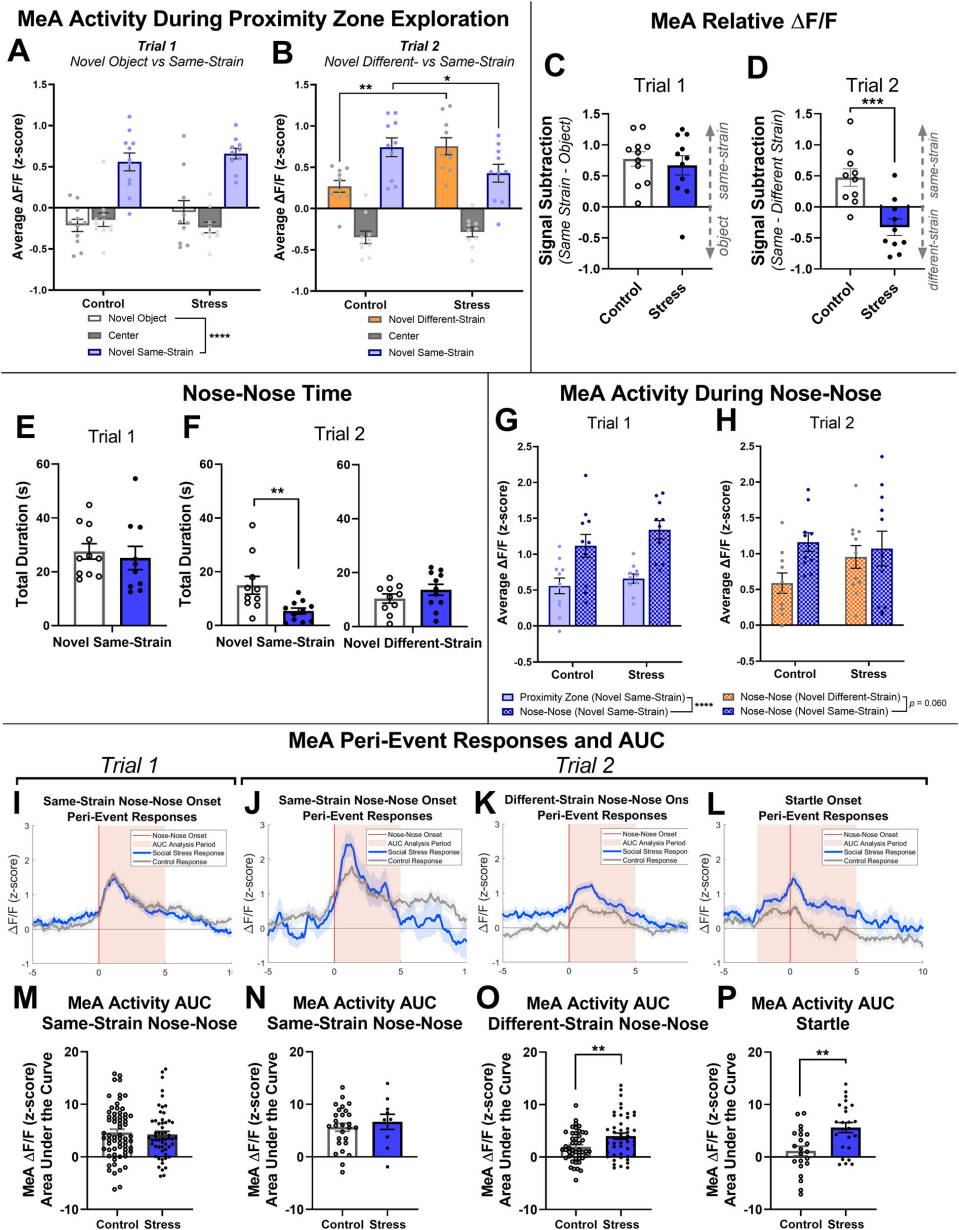
Socially stressed rats showed stimulus-selective MeAp responses. ***A***, In Trial 1 (novel same-strain rat vs novel object), both social stress and control rats showed higher posterior medial amygdala (MeAp) activity while in the novel same-strain rat proximity zone than the novel object proximity zone or center zone. ***B***, In Trial 2 (novel same-strain rat vs novel different-strain rat), socially stressed rats showed lower MeAp activity while in the novel same-strain rat proximity zone and higher MeAp activity while in the novel different-strain rat proximity zone compared with controls. We calculated the relative MeAp Δ*F*/*F* signal for Trial 1 (same-strain–object) and Trial 2 (same-strain–different-strain). ***C***, In Trial 1, relative MeAp activity was similar between social stress and control groups. ***D***, In Trial 2, MeAp activity favored the novel same-strain rat in controls but favored the novel different-strain rat in socially stressed rats. We measured the amount of nose–nose contact between the experimental rat and the novel same-strain rat and novel different-strain rat during social preference tests. ***E***, In Trial 1, the amount of nose–nose contact was similar between social stress and control groups. ***F***, In Trial 2, socially stressed rats engaged in significantly less nose–nose contact with the novel same-strain rat but showed a similar amount of nose–nose contact with the novel different-strain rat, compared with controls. ***G***, Posterior medial amygdala (MeAp) activity was significantly higher in both groups during nose–nose contact with the novel same-strain rat compared with MeAp activity while in the proximity zone in general during Trial 1. ***H***, There was a trend toward higher MeAp activity while engaging in nose–nose contact with the novel same-strain rat compared with the novel different-strain rat; however, there were no differences between social stress and control groups in Trial 2. We illustrated the temporal pattern of MeAp activity in relation to the onset of several behaviors, including (***I***) nose–nose contact with the novel same-strain rat in Trial 1, (***J***) nose–nose contact with the novel same-strain rat in Trial 2, (***K***) nose–nose contact with the novel different-strain rat in Trial 2, and (***L***) startle events while in the novel different-strain rat proximity zone in Trial 2. Perievent MeAp activity was also quantified using the AUC of each event. ***M***, In Trial 1, MeAp activity 0 to 5 s after the onset of nose–nose contact with the novel same-strain rat was similar in socially stressed and control groups. ***N***, In Trial 2, MeAp activity 0 to 5 s after the onset of nose–nose contact with the novel same-strain rat was also similar in both groups. ***O***, However, MeAp activity 0 to 5 s after the onset of nose–nose contact with the novel different-strain rat was higher in socially stressed rats compared with controls. ***P***, MeAp activity 2.5 s before to 5 s after the onset of startle behaviors (red vertical line) near the novel different-strain rat was higher during events in socially stressed rats compared with controls. Data shown as mean ± SEM for each rat in ***A–L*** and mean ± SEM for each event in ***M–P***. *p < 0.05, **p < 0.01, ***p < 0.001.

To quantify changes in MeAp activity during the social preference tests, we used fiber photometry to measure real-time MeAp population activity that was time locked to the behavioral data (Fig. 3*B*). MeAp activity was clearly responsive to social stimuli in both stress and control conditions during Trial 1 (Fig. 3*C*,*E*) and Trial 2 (Fig. 3*D*). In Trial 1 (novel same-strain rat vs novel object), both socially stressed and control rats had higher average MeAp Δ*F*/*F* while in the novel same-strain rat proximity zone compared with the novel object proximity zone (Fig. 4*A*; main effect of zone, *F*_(1,19) _= 55.5, *p *< 0.0001, two-way mixed-effects model ANOVA)^z^. Post hoc testing did not reveal significant differences between stress and control groups in either zone. Therefore, the social stress used here did not influence overall MeAp activity in response to a novel same-strain rat. In Trial 2 (novel same-strain vs novel different-strain rat), socially stressed and control rats had different MeAp Δ*F*/*F* while in the novel same-strain proximity zone and novel different-strain rat proximity zone (Fig. 4*B*; condition × zone interaction, *F*_(1,18) _= 17.2, *p *= 0.0006, two-way mixed-effects model ANOVA, one outlier removed from stress group)^A^. Stressed rats showed higher MeAp activity while in the novel different-strain rat proximity zone (*p *= 0.0031)^B^ and lower MeAp activity while in the novel same-strain rat proximity zone compared with controls (*p *= 0.033, post hoc Holm–Sidak's multiple-comparisons test)^C^. This indicates that social stress reduced the MeAp response to a novel same-strain rat and enhanced the MeAp response to a novel different strain that was the source of prior stress (i.e., a different-strain threat).

### Social stress shifted weight of MeAp response toward different-strain rat

To determine whether MeAp may selectively tune its activity toward certain stimuli, we examined the relative Δ*F*/*F* signal preference by subtracting MeAp activity in the novel object proximity zone (Trial 1) or novel different-strain rat proximity zone (Trial 2) from MeAp activity in the novel same-strain rat proximity zone. This measure more concisely reflects the relative weight of the MeAp response toward either the novel same-strain rat or the novel object/different-strain rat.

In Trial 1 (novel same-strain rat vs novel object), there were no differences in the relative (same-strain–object) MeAp Δ*F*/*F* signal between stressed and control rats (Fig. 4*C*; control: *n *= 11 rats, 0.77 ± 0.12, 95% CI [0.51, 1.0]; stress: *n *= 11 rats, 0.67 ± 0.16, 95% CI [0.32, 1.0]; *t*_(20) _= 0.53, *p *= 0.60, unpaired *t* test)^D^, indicating that both groups responded more strongly to the social stimulus and with equal magnitude. In Trial 2 (novel same-strain vs novel different-strain rat), stressed rats had a lower preference of MeAp Δ*F*/*F* signal toward the same-strain rat than controls (Fig. 4*D*; control: *n *= 10 rats, 0.47 ± 0.14, 95% CI [0.16, 0.79]; stress: *n *= 10 rats, (−0.33) ± 0.13, 95% CI [−0.63, −0.024]; *t*_(18) _= 4.1, *p *= 0.0006, unpaired *t* test, one outlier removed from stress group)^E^. While the MeAp in control rats still responded preferentially to the novel same-strain rat, the MeAp in socially stressed rats responded preferentially to the novel different-strain rat. This aligns with, and provides a potential neuronal correlate for, the shift of behavioral preference in the presence of a different strain that was the prior source of stress.

### Nose–nose behavior elicited the strongest MeAp activity

To understand more about what the differences in MeAp signal may encode, we examined the signal during several behaviors that correspond to strong social interaction using BENTO (Segalin et al., 2021).

In Trial 1 (novel same-strain rat vs novel object), there was no difference between stress and control groups in the amount of time spent engaging in nose–nose interaction with the novel same-strain rat (Fig. 4*E*; control: *n *= 11 rats, 27.6 ± 2.9 s, 95% CI [21.2, 33.9]; stress: *n *= 10 rats, 25.2 ± 4.3 s, 95% CI [15.4, 34.9]; *t*_(19) _= 0.47, *p *= 0.64, unpaired *t* test)^F^, consistent with past research (Li et al., 2017). However, in Trial 2 (novel same-strain rat vs novel different-strain rat), stressed rats spent significantly less time in nose–nose interaction with the same-strain rat than controls (Fig. 4*F*, left; control: *n *= 10 rats, 15.0 ± 3.3 s, 95% CI [7.5, 22.4]; stress: *n *= 11 rats, 5.4 ± 1.1 s, 95% CI [2.9, 7.9]; *t*_(19) _= 2.9, *p *= 0.010, unpaired *t* test)^G^. Both groups spent a similar amount of time engaged in nose–nose contact with the novel different-strain rat (Fig. 4*F*, right; control: *n *= 10 rats, 10.4 ± 1.7 s, 95% CI [6.6, 14.2]; stress: *n *= 11 rats, 13.6 ± 2.0 s, 95% CI [9.1, 18.2]; *t*_(19) _= 1.2, *p *= 0.24, unpaired *t* test)^H^. These results suggest that the presence of the novel different-strain rat decreased both time spent near the novel same-strain rat, as described in Figure 2, as well as time spent engaging in close nose–nose contact with a novel same-strain rat.

MeAp activity was significantly higher in both groups during nose–nose contact with a novel same-strain rat compared with MeAp activity while in the proximity zone in general during Trial 1 (Fig. 4*G*; main effect of zone, *F*_(2,38) _= 76.7, *p *< 0.0001, two-way mixed-effects model ANOVA)^I^, illustrating that MeAp activity is more specific for close social contact rather than simple proximity to a social stimulus. We then compared MeAp activity elicited by nose–nose contact with the two different strains of rats in Trial 2 between stress and control groups. There was a trend toward higher MeAp activity while engaging in nose–nose contact with the novel same-strain rat compared with the novel different-strain rat (Fig. 4*H*; main effect of zone, *F*_(1,38) _= 3.7, *p *= 0.060, two-way mixed-effects model ANOVA)^J^, but no significant difference between stress and control groups. However, when examining how the MeAp responded in real time to the onset of social behaviors, we could clearly observe increases in the MeAp activity following the onset of nose–nose contact with a same-strain rat in Trial 1 (Fig. 4*I*), the novel same-strain rat in Trial 2 (Fig. 4*J*), and the different-strain rat in Trial 2 (Fig. 4*K*). We additionally observed high levels of MeAp activity in Trial 2 when the different-strain (Long–Evans) rat attempted to bite or paw strike through the bars of the cage, producing a defensive startle response (Fig. 4*L*). To capture the magnitude MeAp activity more accurately during these social events, the peri-event MeAp activity was quantified using the AUC in the time period of the onset of each observed behavioral event. In Trial 1 (novel same-strain rat vs novel object), there was no difference between stress and control groups in the amount of MeAp Δ*F*/*F* activity after initiating nose–nose contact with the novel same-strain rat (AUC of initial 5 s during nose–nose bout; Fig. 4*M*; control: *n *= 64 events, 4.7 ± 0.6, 95% CI [3.4, 5.9]; defeat: *n *= 56 events, 4.3 ± 0.6, 95% CI [3.0, 5.5]; *U *= 1691, *p *= 0.60, Mann–Whitney *U* test)^K^, indicating that socially stressed and control rats responded with a similar degree of MeAp activity during nose–nose contact with a novel same-strain rat. In Trial 2 (novel same-strain rat vs novel different-strain rat), there was again no difference between stress and control groups in the amount of MeAp Δ*F*/*F* activity after initiating nose–nose contact with the same-strain rat (AUC of initial 5 s during nose–nose bout; Fig. 4*N*; control: *n *= 27 events, 5.6 ± 0.8, 95% CI [4.1, 7.2]; stress: *n *= 10 events, 6.7 ± 1.4, 95% CI [3.4, 9.9]; *t*_(35) _= 0.67, *p *= 0.51, unpaired *t* test)^L^. However, stressed rats had significantly higher MeAp Δ*F*/*F* activity than controls after initiating nose–nose contact with the different-strain rat (AUC of initial 5 s during nose–nose bout; Fig. 4*O*; control: *n *= 46 events, 1.9 ± 0.4, 95% CI [1.1, 2.8]; defeat: *n *= 47 events, 4.0 ± 0.6, 95% CI [2.8, 5.2]; *t*_(91) _= 2.8, *p *= 0.0065, unpaired *t* test)^M^. Of note, the MeAp Δ*F*/*F* activity during the baseline period (−5 to 0 s) prior to the onset of nose–nose contact with the different-strain rat was higher in socially stressed rats than controls. We attribute this to higher MeA activity while approaching the proximity zone of the different-strain rat, even before nose–nose contact had been initiated. Overall, MeAp responses after stress were preferential toward conditions where a social threat was present and aligned with a shift in social behaviors. These results indicate that the MeAp strongly encodes a social signal, but this signal is sensitive to the nature of the social partner, particularly to whether prior experience indicates that it may be a threat.

The startle response to an aggressive behavior produced by a different-strain rat is readily interpretable as defensive and can yield useful information about encoding by the MeAp. Stressed rats had significantly higher MeAp Δ*F*/*F* activity than controls in the time period surrounding the defensive response triggered by the aggressive different-strain behavior (2.5 s before to 5 s after initiation of defensive response; Fig. 4*P*; control: *n *= 22 events, 1.1 ± 0.9, 95% CI [−0.8, 3.0]; stress: *n *= 24 events, 5.6 ± 1.0, 95% CI [3.6, 7.6]; *t*_(44) _= 3.4, *p *= 0.0015, unpaired *t* test)^N^. This indicates that rats with prior experience of being attacked by this different-strain exhibited a higher MeAp response to the aggression of the different-strain rat. Lastly, although our study was not specifically designed to test for laterality in the brain, there were no systematic differences between right and left sides in the MeA response to novel object, same-strain or different-strain stimuli.

## Discussion

The MeA encodes social cues that have motivational significance (Newman, 1999; Choi et al., 2005; Hong et al., 2014; Wang et al., 2014; Shemesh et al., 2016; Li et al., 2017; Aleyasin et al., 2018). Here, we show that a novel same-strain rat can activate the MeAp during social investigation, and this is partially attenuated if a rat has experienced social stress. The MeAp weakly responded to a different-strain rat, unless there was prior aversive experience with that strain. This demonstrates that the MeAp does not indiscriminately encode conspecific social cues and that prior social stress can impact MeAp encoding in a strain-specific manner.

We observed increased social investigation of a novel same-strain rat at the beginning of the test in stressed rats relative to control in Trial 1, which was surprising given other studies that report social avoidance following social defeat (Berton et al., 2006; Tsankova et al., 2006; Hollis et al., 2010; Razzoli et al., 2009, 2011; Vidal et al., 2011; Hollis and Kabbaj, 2014; Tian et al., 2018; Munshi et al., 2020). However, other studies have also shown paradoxical increases in social approach following social stress (Nelson et al., 2010; Cumming et al., 2014; Boyson et al., 2016). The heightened same-strain social approach may therefore represent learned safety of the same-strain. Consistent with this, the heightened approach to same-strain rats in Trial 1 became similar to control rats after the initial 2 min. This may be due to the stressed rats having the opportunity to sample the environment for threats and assess that there was no different-strain aggressor present.

A novel finding was the manner in which the behavioral response to the same-strain rat was shifted in the presence of the different-strain rat, but only if there was prior negative experience with that different strain. Experienced rats showed less interaction with the novel same-strain rat compared with controls, indicating that the presence of the different-strain rat was sufficient to shift experienced rats from increased social investigation of same-strain rats toward social avoidance. Although stressed rats were also less interactive with different-strain rats compared with control rats, both groups showed a persistent preference for the different-strain rat. It was not expected that rats with negative experience with the different strain would display behavioral preference for the different-strain rat over the same-strain rat. However, this unusual behavior does fit with some prior studies across rodents (Potegal et al., 1993; Nelson et al., 2010; Cumming et al., 2014; Boyson et al., 2016) and may not reflect a positive valenced preference but rather that the rat was in a defensive state, awaiting attack. This is consistent with defensive postures observed after resident–intruder stress during re-exposure to the context where attack occurs or the aggressive strain itself (Frischknecht et al., 1982; Tornatzky and Miczek, 1994; Meerlo et al., 1999; Walker et al., 2008; Wood et al., 2010). This behavioral preference was maintained in the stress group throughout the session, perhaps indicating an enduring negatively valenced affective response to the different strain based on prior negative experience, while controls gradually shifted to a neutral preference.

Despite demonstrating a prolonged behavioral preference toward the different-strain rat, the stressed rats also displayed fragmented social behavior in the presence of that different-strain rat, which we observed as shorter bout duration and increased number of transitions between stimuli. Therefore, stress-related social investigation consisted of periods of repeated quick sampling of the environment and retreating. The presence of a threatening social cue may create an aversive, fearful social context that increases the level of uncertainty in previously stressed rats. Overall, these results suggest that socially stressed rats showed persistent caution and uncertainty, observed as heightened social approach toward a same-strain conspecific when the threat was absent, and as fragmented social behavior and persistent monitoring of the threat when present, at the expense of investigation of the same-strain conspecific. These behaviors in stressed rats may be interpreted as adaptive behaviors toward a previously experienced threat.

These behavioral outcomes provided a unique set of conditions to test how same- and different-strain individuals are processed by the MeAp, and how this is modulated by prior negative experience with the different strain. The results first replicated the high sensitivity of the MeAp to same-strain conspecifics (Li et al., 2017). It was mildly surprising that prior social stress did not impact MeAp response to the same-strain rat in Trial 1, when the aggressive different-strain rat was absent. However, in the context of the current social stress paradigm and behavioral results, this may be expected. In the resident–intruder approach used here, stress was imposed entirely by a different-strain rat in a specific context of that different-strain home cage. Indeed, as demonstrated by social behavior, prior social stress only subtly impacted the behavioral response to a novel same-strain rat.

Prior work has measured MeAp immediate early gene activation by threats from a different species, such as predators, same-strain conspecific threats, and different-strain conspecific threats (Kollack-Walker and Newman, 1995; Martinez, 1998; Dielenberg et al., 2001; Covington et al., 2005; Masini et al., 2005; Veening et al., 2005; Staples et al., 2008; Fekete et al., 2009; Samuelsen and Meredith, 2009; Fanous et al., 2010; Butler et al., 2011; Martinez et al., 2011; Wohleb et al., 2011; Morrison et al., 2012; Weathington et al., 2012; Lkhagvasuren et al., 2014), and compared MeA activity between conspecific and predator odors (Bergan et al., 2014). Our results indicate that real-time MeAp responsiveness is highly dependent on the stimulus and prior experience. While both socially stressed and control rats showed high MeAp activity while investigating a novel same-strain rat, there was a relatively weak MeAp response to a different-strain rat in controls, perhaps reflecting its differing social significance in inexperienced rats. This suggests that MeAp discriminately responds to social threats, with enhanced MeAp response toward the different strain following negative experience, at the expense of the MeA response to same-strain rats. The shift in MeAp activity preference toward threatening social cues following stress experience was mirrored in the behavioral preference for the strain that was the source of prior stress. Heightened MeAp activity was also closely associated with both the onset of nose–nose interaction with the different-strain rat and defensive responses triggered by aggressive behavior of the different strain, in a way that was enhanced following social stress, suggesting that the MeAp may drive heightened reactivity to social threats.

Based on the results presented here, the MeA may play a role in both positive and negative social interactions. Heightened MeA activity accompanied both social approach toward a novel same-strain rat in a safe environment, as well as generalized avoidance and fragmented social behavior in the presence of a social threat. These findings extend the known role of the MeAp in detection of many types of social stimuli and support a role of the MeAp in biasing toward the detection of important social stimuli with robust significance, and tuning of that importance based on prior experience.

There are several limitations to this study. First, the study was limited to male rats. While social defeat stress produces robust effects in male rats, feasibility issues exist when replicating this model in female rats. Alternative approaches for female social stress (e.g., witness stress or attacks by lactating rats) had less robust effects in our hands but should be explored in future experiments. Second, Trial 1 and Trial 2 were not counterbalanced, to avoid possible effects of stressor re-exposure on a subsequent trial. While it is possible that there were time-dependent effects in the present study, this would be largely mitigated by comparisons between the stress and control groups that had the same trial orders. The different-strain (Long–Evans) rats used in the study were older and larger in size than the experimental rats, which is an established paradigm for resident–intruder stress. While older rats interact less than younger rats (Salchner et al., 2004), in the present study the social interaction was initiated entirely by the experimental rats. However, it is possible that the age or size of the different-strain (Long–Evans) rat may have contributed to the experimental rat behavior in Trial 2 of the social preference test. Additionally, we identified high MeAp activity during nose–nose contact post hoc. Due to the cylindrical shape of the cages in the preference apparatus, there were small portions of the chamber that were not visible, and there were likely a few nose–nose contacts that were occluded from view. Therefore, we scored this behavior conservatively, but this will be refined in future experiments, as it may yield higher and more specific changes in MeAp activity. Last, our study cannot determine whether the changes in MeAp activity caused the observed changes in social behavior. Future studies should selectively activate or inactivate the MeAp and its functional pathways to conclusively determine the function of specific MeAp circuits in driving the effects of social stress on these social behaviors.

### Conclusions

Our study shows that the MeAp is sensitive to social stress, but in a manner that discriminates between social stimuli in the environment. There is good prior evidence that the MeA is a critical node for integrating information about the social environment. The MeA integrates social cues via olfactory centers (Maaswinkel et al., 1996; Ferguson et al., 2001; Gur et al., 2014) with environmental information from the BLA (Markham and Huhman, 2008) to produce situation-dependent social responses and assist in threat discrimination. The results here present a novel reformulation of the role for the MeAp in social engagement. While much prior work established a sensitivity of the MeAp to conspecific and predator odors (Masini et al., 2005; Staples et al., 2008; Samuelsen and Meredith, 2009; Wu et al., 2009; Been and Petrulis, 2011; Butler et al., 2011; Martinez et al., 2011; Bergan et al., 2014; Li et al., 2017; Yao et al., 2017; Kikusui et al., 2018), real-time activity provides a more subtle interpretation. The MeAp has a preference for same-strain conspecifics instead of different-strain conspecifics at baseline. But, the MeAp does not robustly respond to social odors with low salience, such as unfamiliar different-strain conspecifics (Papes et al., 2010; Sánchez-Juanes et al., 2013; Kashiwayanagi, 2014; Gómez-Baena et al., 2019; Nikaido, 2019). Instead of responding in a “stranger danger” mode, MeAp appears to respond to strangers in a manner that is specific to prior experience. Given the role of the MeAp in a range of social behaviors, from social engagement to reproductive behaviors (Newman, 1999; Choi et al., 2005; Hong et al., 2014; Wang et al., 2014; Shemesh et al., 2016; Li et al., 2017; Aleyasin et al., 2018), this would imply that MeAp circuitry is wired to drive social or defensive behavior toward same-strain conspecifics, but this can be shifted toward different-strain conspecifics at the expense of same-strain conspecifics based on experience.

## Synthesis

Reviewing Editor: Leandro Vendruscolo, National Institute on Drug Abuse Decisions are customarily a result of the Reviewing Editor and the peer reviewers coming together and discussing their recommendations until a consensus is reached. When revisions are invited, a fact-based synthesis statement explaining their decision and outlining what is needed to prepare a revision will be listed below. The following reviewer(s) agreed to reveal their identity: NONE.

Reviewer 1:

This manuscript focuses on the effect of social stress on subsequent social interaction with either the same or a different strain of rats and its effect on the activity of the posterior medial amygdala (pMeA). After inducing social stress, the authors used a three-chamber social preference test. They demonstrated that in different trials, rats that experienced social stress preferred to spend time with a same-strain rat over a novel object (trial 1). The study showed initially heightened social interaction with same-strain rats and potential social avoidance with different-strain rats compared to controls. Additionally, pMeA activity, assessed using fiber photometry, was similarly activated during interactions with a same-strain rat for both stressed and control rats. There was a potential increase in pMeA activity for stressed rats in trial 2 when interacting with different-strain rats. This paper addresses an important research question in social neuroscience and could contribute to future investigations on the role of the medial amygdala (MeA) in negative and positive social behaviors.

Comment to the authors:

- The manuscript is not easy to follow and is particularly lengthy in several sections. The authors should consider reorganizing the manuscript to enhance clarity and readability. For instance, the results and discussion of the first three figures could be combined into a single figure with a dedicated result section summarizing the findings. This consolidation is suggested because the data presented in these figures re-analyze the same results in different ways. Similar comments apply to Figures 4 and 5. Additionally, please revise the introduction and discussion sections to reduce the overall length of the manuscript.

- Social stress results should be presented with dedicated summary graphs and diagrams or timelines of the events.

- Please consider revising the presentation of Figure 1, as it is currently difficult to read.

- Please specify if the two different trials were counterbalanced to ensure that both behaviors and photometry results were not temporally biased.

- The potential social avoidance and 'fragmented' social behaviors towards the different-strain rats need to be quantified and shown with dedicated graphs.

- In the methods section, the authors reported a histology paragraph, but no representative images are presented. Please add representative images of viral expression for all conditions.

- Due to the descriptive nature of this manuscript, it is challenging to draw conclusions regarding the role of MeA in either negative or positive social interactions. The authors should discuss this point and propose a potential role of MeA in social stress and interactions based on their photometry results.

Reviewer 2:

The findings suggest that increased activity in the MeAp region of the brain may influence how rats respond socially to novel and potentially threatening stimuli following stress. The methods of the study are appropriate to investigate the phenomenon.

I would like the authors to comment on the age of the rats used (same-strain vs different-strain). Are they the same age? Is there any concern in social interaction between rats at different ages?

MeA activity was recorded in a side counterbalance way. Are the findings consistent between different sides? Was any lateralization observed?

Figure 3B should show a statistical main effect

Figure 4 (C,D,E and F) should be reorganized with controls on the left (or top) and stress on the right (or bottom). The legend should be fixed.

Figure 6 C should show a statistical main effect

Figure 6 G shows differences between groups before nose-nose onset. Please discuss.

Figure 6 H legend red line should be startle onset.
